# Retraction: Human Phosphatidylethanolamine-Binding Protein 4 Promoted the Radioresistance of Human Rectal Cancer by Activating Akt in an ROS-Dependent Way

**DOI:** 10.1371/journal.pone.0210896

**Published:** 2019-01-10

**Authors:** The PLOS ONE Editors

Following publication of this article [1], concerns were raised regarding the following figure panels:

- Fig 1C hPEBP4 panel lane 1 and Fig 4F p-Akt lane 3 appear similar.
- Fig 1C hPEBP4 panel lane 2 and Fig 4F p-Akt lane 2 appear similar.
- Fig 2A hPEBP4 panel: there appears to be a vertical line suggestive of image splicing between lanes 1 and 2.
- Fig 2D hPEBP4 panel: there appears to be a vertical line suggestive of image splicing between lanes 1 and 2.
- Fig 2D B-actin panel: there appears to be a vertical line suggestive of image splicing between lanes 3 and 4.

The authors explained that they outsourced experiments reported in this article to four external companies. The involvement of external companies was not declared in the article. The authors were unable to provide an explanation for the concerns raised and do not have access to the full raw data underlying any figures in this article.

In light of these concerns, the *PLOS ONE* Editors retract this article.

JQ, GY, AL, DW, LD did not respond. ZS could not be reached.

## References

[1] Qiu J, Yang G, Lin A, Shen Z, Wang D, Ding L (2014) Human Phosphatidylethanolamine-Binding Protein 4 Promoted the Radioresistance of Human Rectal Cancer by Activating Akt in an ROS-Dependent Way. PLoS ONE 9(3): e90062 10.1371/journal.pone.0090062 24594691 PMC3940727

